# Retinal Wave Behavior through Activity-Dependent Refractory Periods

**DOI:** 10.1371/journal.pcbi.0030245

**Published:** 2007-11-30

**Authors:** Keith B Godfrey, Nicholas V Swindale

**Affiliations:** Department of Opthamology and Visual Sciences, University of British Columbia, Vancouver, British Columbia, Canada; University College London, United Kingdom

## Abstract

In the developing mammalian visual system, spontaneous retinal ganglion cell (RGC) activity contributes to and drives several aspects of visual system organization. This spontaneous activity takes the form of spreading patches of synchronized bursting that slowly advance across portions of the retina. These patches are non-repeating and tile the retina in minutes. Several transmitter systems are known to be involved, but the basic mechanism underlying wave production is still not well-understood. We present a model for retinal waves that focuses on acetylcholine mediated waves but whose principles are adaptable to other developmental stages. Its assumptions are that a) spontaneous depolarizations of amacrine cells drive wave activity; b) amacrine cells are locally connected, and c) cells receiving more input during their depolarization are subsequently less responsive and have longer periods between spontaneous depolarizations. The resulting model produces waves with non-repeating borders and randomly distributed initiation points. The wave generation mechanism appears to be chaotic and does not require neural noise to produce this wave behavior. Variations in parameter settings allow the model to produce waves that are similar in size, frequency, and velocity to those observed in several species. Our results suggest that retinal wave behavior results from activity-dependent refractory periods and that the average velocity of retinal waves depends on the duration a cell is excitatory: longer periods of excitation result in slower waves. In contrast to previous studies, we find that a single layer of cells is sufficient for wave generation. The principles described here are very general and may be adaptable to the description of spontaneous wave activity in other areas of the nervous system.

## Author Summary

Neurons from the immature retina extend axons that make connections in the visual centers of the brain. Chemical markers provide guidance for these axons, but patterned neural activity is necessary to refine their connections. Much of this activity occurs in a distinctive pattern of waves before the retina is responsive to light, but it is not known how these waves are generated. In this study, we describe a simple mechanism that can explain the production of retinal waves. We use the knowledge that immature retinal cells are spontaneously active and show that waves will result if cells that receive more input when they are spontaneously active have longer intervals between activity. The resulting model reproduces experimentally observed waves in a variety of species, including ferret, chick, mouse, rabbit, and turtle, both at the level of individual cells and of the entire retina. The behavior appears intrinsically chaotic and the model is not tied to the properties of any particular biochemical pathway. We suggest that this mechanism could underlie not only the spontaneous patterns of activity that are generated in the retina but other areas of the developing brain as well.

## Introduction

In the early stages of neural development, when initial sets of connections between neurons are being formed, neural activity helps organize and refine developing circuits. Before the onset of stimulus-driven activity, which helps refine neural organization in later developmental stages, neural circuits generate spontaneous patterns of activity which guide early development [1]. This spontaneous activity has been observed in many areas of the developing nervous system, including the auditory system [2,3], neocortex [4], hippocampus [5], spinal cord networks [6,7], brainstem nuclei [8], and retina [9,10]. In the retina, spontaneous activity takes the form of coordinated bursts of spikes in neighboring retinal ganglion cells (RGCs) that slowly spread across the retina [10,11]. Retinal waves occur in a variety of species before visual experience, including cat, turtle, chick, mouse, and ferret [12]. They have non-repeating boundaries [13,14], propagate with no directional bias, and can initiate at any retinal location [10,11,13,15]. The entire retina is covered in minutes [13,14,16].

Retinal waves drive activity-dependent organization in the visual system [1,11,12,17]. They have been shown to refine retinotopy in the LGN, superior colliculus, and cortex [18–25], to drive segregation of the LGN into eye-specific layers [17,19,22], and to drive responses in V1 neurons [26]. While the physiological mechanisms underlying retinal waves have been studied extensively [12,17,27], there have been few attempts at modeling them. The first model was based on extracellular diffusion of potassium driving RGC activity [28]. Experimental evidence contradicted this premise [13] and another model was put forward, based on random amacrine cell activity and long refractory periods where amacrine cells are non-responsive [14]. Subsequent physiological evidence has shown these assumptions to be invalid, as amacrine cells regularly depolarize during waves and release excitatory transmitter when doing so [29,30]. Other limitations are that the model produces non-uniform net coverage of the retina [31], that it has only been demonstrated to produce waves similar to postnatal day 2 (P2) to P4 ferret, and that the properties of the generated waves, including wave size, frequency, and velocity, can be very sensitive to small changes in network state or parameters [32].

In this study we describe a retinal wave model that is robust to parameter variation and generalizes across species. We make use of the findings that amacrine cells receive input and depolarize during local wave activity [13,29,30], that they have variable periods between spontaneous depolarizations [29,30], and that the period between depolarizations appears to be a function of recent local excitation [30]. These observations lead to the central principle behind the model, that the refractory period, or the period between spontaneously occurring bursts in cells, is a function of the amount of excitatory input recently received by the cell. The resulting model produces waves with randomly distributed initiation points and non-repeating borders across a wide range of parameter settings. The velocity, domain size, and interwave interval (IWI) of the generated waves can be configured to match those seen in ferret, rabbit, mouse, turtle, and chick retinas. We show that a single homogenous group of cells can produce these wave behaviors, in contrast to claims that such behavior requires two independently functioning cell types [14]. We also show that the model exhibits chaotic behavior, producing seemingly random patterns of waves in the absence of stochastic input. The uniformity of retinal coverage provided by the model and the realistic spatio–temporal patterns of activity should also make it useful as an input to developmental models of the retino–geniculo–cortical pathway [31,33].

## Results

### Ferret Waves (P2–P4)

**Figure 1:**
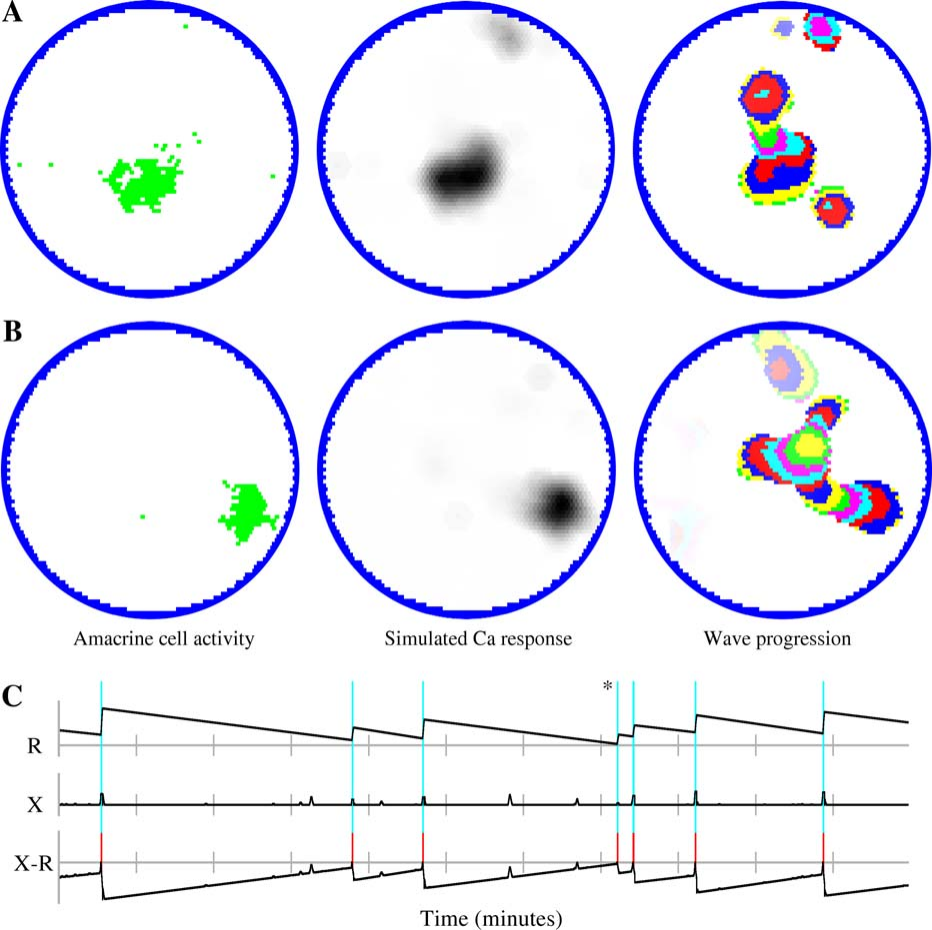
Examples of Wave Behavior (A,B) Left circles show the instantaneous states of amacrine cells at two different times in the same retina. Resting cells are white, and depolarized cells are green. Center circles show simulated responses seen with calcium imaging. Circles on the right show retinal activity over the proceeding 15 s. Different colors show the progression of waves during 500 ms intervals. Colors fade to white with time. Widths of the different color bands show variations in the velocity of the advancing wave front. (C) The behavior of threshold and excitation in a randomly selected amacrine cell over 11 min simulated time. The threshold plot (R*:* black lines) shows slow, linear decay with periodic increases coinciding with the depolarization of the cell (vertical blue lines). The magnitude of threshold increase varies as a function of input received by the cell when it is depolarized. Large threshold increases generally indicate that the cell is in a central and fast-moving portion of a wave. Smaller threshold increases occur when a cell is in a slow-moving part of a wave or near a wave boundary, or when it depolarizes in the absence of a wave. The excitation plot (X) shows input to the cell due to nearby wave activity. This input is sometimes enough to overcome threshold and cause the cell to depolarize. The third graph (X-R) shows the difference between excitation and threshold levels. The cell fires when excitation exceeds threshold (red line segments). This plot resembles voltage recordings in amacrine cells [30], consistent with an AHP playing a large but not exclusive role in the threshold change. Peak levels of excitation are small compared to threshold levels. Most amacrine cell depolarizations are the result of nearby wave activity with approximately 10% occurring “spontaneously” (*).

The waves produced by the model are qualitatively comparable to published images of physiological waves [13,14,34,35]. Figure 1 shows two examples of wave activity and Video S1 shows 4 min of simulated wave behavior. Waves begin when several nearby amacrine cells are at or near the point of spontaneous depolarization and the excitation produced by the depolarization of some cause premature depolarization in others. If a sufficiently high density of depolarized amacrine cells is present, a wave develops. The wave continues to propagate in all directions where there is a high enough density of amacrine cells capable of depolarizing as a function of the excitatory input from their neighbors.

**Figure 2:**
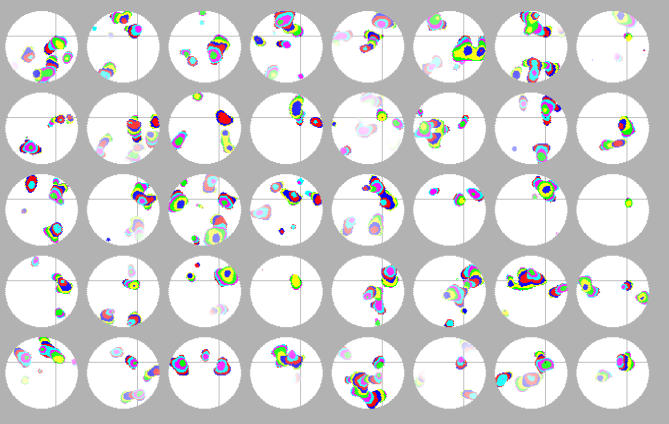
History of Waves Passing through a Single Point Snapshots of 40 successive waves that passed through a randomly selected point on the retina (identified by gray cross-hairs). Images are ordered left to right, top to bottom, and show the entirety of this wave as well as all concurrent activity. Different colors show the progression of waves during 500 ms intervals and colors fade to white with time (15 s). These images were produced when the simulation was run in deterministic mode (i.e., using no “noise”), and they show no indication of cyclic or repeating activity over the observation interval. Instead, waves through a given point, and activity across the entire retina, appear to be random.

The non-repeating wave behavior occurs because amacrine cells receive differing amounts of input during wave activity, resulting in some cells becoming more refractory than others. Figure 1C shows the threshold and excitation of a cell over time. Amacrine cells near the central regions of a wave receive more input during a wave, and hence become more refractory, than amacrine cells near the wave boundaries. This provides a deterministic and destabilizing force that inhibits the production of repeating wave domains as subsequent waves will more readily “invade” the border areas of a previous domain than central areas. Observations have shown that both the form of the original domain and the timing of these “invasions” determine the extent of the invasion and the subsequent increase in refractoriness for the amacrine cells involved. This turns the largely coherent refractoriness of amacrine cells in the original domain into several incoherent subgroups, inhibiting generation of a subsequent wave capable of following the boundaries of a predecessor. Figure 2 shows 40 sequential waves passing through a randomly selected spot on the retina, giving an example of the variability and non-repeating quality of the waves.

**Figure 3:**
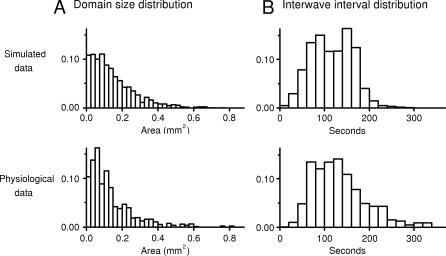
Comparison between Model and Ferret Wave Data (A) The distribution of wave sizes (also called domains [13]) of the model (top) and physiological data (bottom); bin size is 0.025 mm^2^; physiological data are the average of five P2–P4 ferrets, data adapted from [14]; model data are based on 3 h of simulated time. All distributions were normalized to 1.0. (B) The IWI distribution of the model (top) and physiological data (bottom), data adapted from [14]. Bin size is 20 s. Table 1A gives the simulation parameters used to produce this output.

The domain size and IWI distributions of simulated and physiologically recorded waves are shown in Figure 3. Figure 3A shows the distribution of domain sizes and the averaged response of five P2–P4 ferret retinas (data from [14]). The two distributions are very similar. Simulated domains average 0.156 ± 0.141 mm^2^ (median 0.119 mm^2^; the average size of physiological domains was not reported). The IWI is defined as the period of time between successive waves passing a given location on the retina. Figure 3B shows plots of IWI distributions measured in the model retina and that are observed experimentally [14]. The model's IWI averaged 117 ± 47 s (median 116 s) which is similar to the experimentally measured value of 115 ± 48 s [13]. As with domain sizes, the model and physiological IWI statistics, and the shapes of the IWI distribution, were very similar. Experimentally reported velocities averaged 177 μm/s with a frequency of 3 waves/mm^2^/s [14]. Corresponding figures for the model were 176 μm/s and 3.0 waves/mm^2^/s.

**Table 1:**
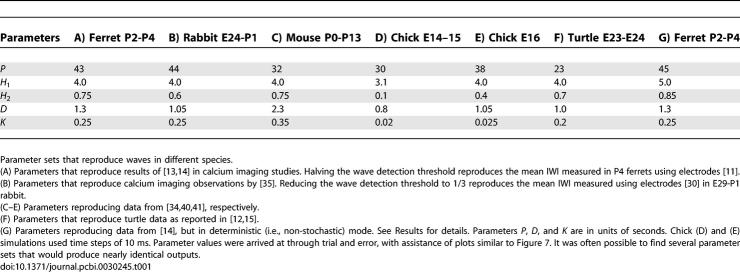
Parameter Sets

The IWI distribution measured by [13] was based on calcium imaging while other studies of similarly aged ferrets [11] used electrodes and reported notably shorter IWIs (90 versus 115 s). If RGC spiking, and hence wave activity, were to occur when amacrine cells were active but below the threshold of detection through calcium imaging, that would explain this discrepancy. Indeed, it has been estimated that RGCs which spike less than seven times in a burst may not be detected in calcium imaging studies [36]. To test this, we halved the wave detection threshold in the model. This reduced the average IWI to 86 ± 43 seconds, producing waves with an average velocity of 162 μm/s. This is comparable to electrode recordings of waves that showed an IWI of 90 s and a velocity of 100–300 μm/s [11]. These results suggest that wave activity and RGC spiking occurs in areas of the retina not detected by large-scale calcium imaging.

**Figure 4:**
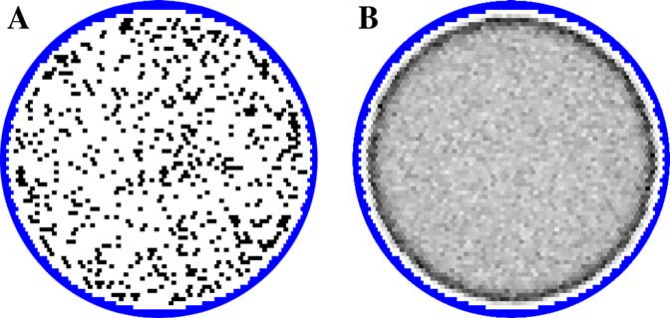
Distribution of Wave Initiation Points (A) Initiation points for 653 waves (60 min simulated time). Each dot shows the initiation point of exactly one wave, with waves sharing the same location being represented with adjacent dots. Points are uniformly distributed, consistent with physiological studies [14,15]. (B) Density plot of wave initiation points after 79,216 waves (120 h simulated time). Shades of gray indicate number of initiation points at each location on the retina with darker colors representing higher activity. Shades are linearly scaled. There is a clear bias for waves to initiate near the retinal boundary.

Physiological studies have reported that waves have a random distribution of initiation points [14–16]. Figure 4A shows the distribution of wave initiation points from the model over a 60 min period. With the exception of the edges, initiation points are distributed uniformly across the retina, consistent with physiological studies [14,15]. Figure 4C shows a density plot of initiation points produced by the model after 120 h simulated time. Border effects are apparent near the retinal boundary, with each point on the boundary having two to three times the rate of wave initiation as a point in the central area. The model does not represent changing densities of amacrine cells in the peripheral and border areas of the retina, a simplification that might create altered patterns of initiation points compared to the physical retina. Physiological retinal wave studies have not yet addressed possible border effects on wave generation.

One application of retinal wave models is for use in modeling development of the retino–geniculate pathway, such as done by Elliott and Shadbolt [31]. Their model required wave activity to be relatively uniform across the retina, as areas of high relative activity would achieve disproportionately large representation in the LGN. In their study they used the most accurate existing retinal wave model [14] and found that it had significant non-uniformities, including areas of high relative activity they termed “hot spots”. The duration of activity of RGCs in the model averaged 96.8 ± 25.4 s after a period of simulation. While individual cells have been reported to have different firing rates [11], experimental studies have not reported observations of “hot spots” or other clear non-uniformities in the spatial variability of retinal activity. A test for the current model was to determine how evenly wave activity was distributed across the retina. We found that each location on the retina was active for an average of 95.8 ± 3.9 s over a 110 min simulation, with no spatial groupings of above or below average activity. There were no suggested acceptable limits for variability by [31], but the reduction in the standard deviation from 26.2% of the mean to 4.1% over a nearly identical duration of activity should greatly improve the uniformity of retinotopic organization in such models and may be more representative of actual retinal behavior.

### Reproduction of Wave Statistics in Different Species

#### Rabbit.

In E24-P1 rabbit, the nicotinic acetylcholine (ACh) system is the primary driving force for retinal waves [37]. During this period, calcium imaging studies have shown waves to have an IWI of 113 ± 25 and a velocity of 200 μm/s [35]. Using the parameters of Table 1B, the model produced an IWI distribution of 112 ± 42 s and a velocity of 199 μm/s. Physiological wave sizes for rabbits have not been published. When not constrained by wave size, several parameter sets can produce waves of this approximate distribution (this particular parameter set produced waves with average domain sizes of 0.19 ± 0.17 mm^2^). As in ferret, electrode recordings in rabbit have shown shorter IWIs compared to measurements done with calcium imaging. As recorded by electrodes, RGCs in E29-P1 rabbits have IWIs of 70 ± 26 s with a median of 64 s [30]. Lowering the calcium detection threshold to 1/3 while keeping all other parameters constant produced an IWI distribution of 74 ± 39 s with a median of 68 s, again consistent with the idea that calcium imaging is not able to detect all RGC activity during waves.

#### Mouse.

Retinal wave velocities in P1–P13 mice averaged 110 μm/s [34], considerably slower than in other species [12]. Using the parameters shown in Table 1C, waves averaging 108 μm/s were produced, with IWI distributions of 82.2 ± 34.8 s and domain sizes averaging 0.19 ± 0.19 mm^2^. This compares with physiological IWI measurements of 83.6 ± 32.3 s and sizes of 0.19 ± 0.21 mm^2^. Other studies [38,39] have reported wave velocities in mice that are notably higher. We elected to target the lower velocities in order to analyze the flexibility of the model. The model is also able to reproduce the higher velocity waves (unpublished data).

#### Chick.

E14–E15 chicks are reported to have waves with velocities of 0.516 ± 0.118 mm/s and an IWI of 95.7 s [40]. These velocities are considerably faster than waves observed during Ach-mediated waves in mammals. The parameters in Table 1D produce waves with velocity 0.525 ± 0.160 mm/s with an IWI of 99 ± 35 s. Another study reported that waves in E16 chicks have average velocities ranging from 0.5 to 1.5 mm/s and that they are very large and originate at different points on the retina [41]. The parameters in Table 1E reproduce these findings. The average wave velocity was 0.856 mm/s. Initiation points were distributed across the simulated retina, similar to Figure 3B, but with more clustering near the borders (unpublished data). Average domain size was 0.91 ± 0.55 mm^2^ (median 0.83 mm^2^). The physiological IWI at this age is reported to be less than 2 min; the IWI of the model was 82 ± 23 s.

#### Turtle.

S23–S24 turtles have waves averaging 226 μm/s [15] with an IWI of 35–90 s [12], faster and more frequent than observed in the mammalian species considered here. Table 1F lists the parameters to produce waves with a velocity of 223 ± 47 μm/s and an IWI of 63.5 ± 24.4 s. While the waves in turtles are rich and well-studied [15,42], we did not attempt to investigate or represent individual neurotransmitter pathways in this or any other species. Modeling waves in turtle, chick, and mouse, which use glutamate during some or all of the ages modeled, was done to show the flexibility of the model.

### Chaotic Behavior

**Figure 5:**
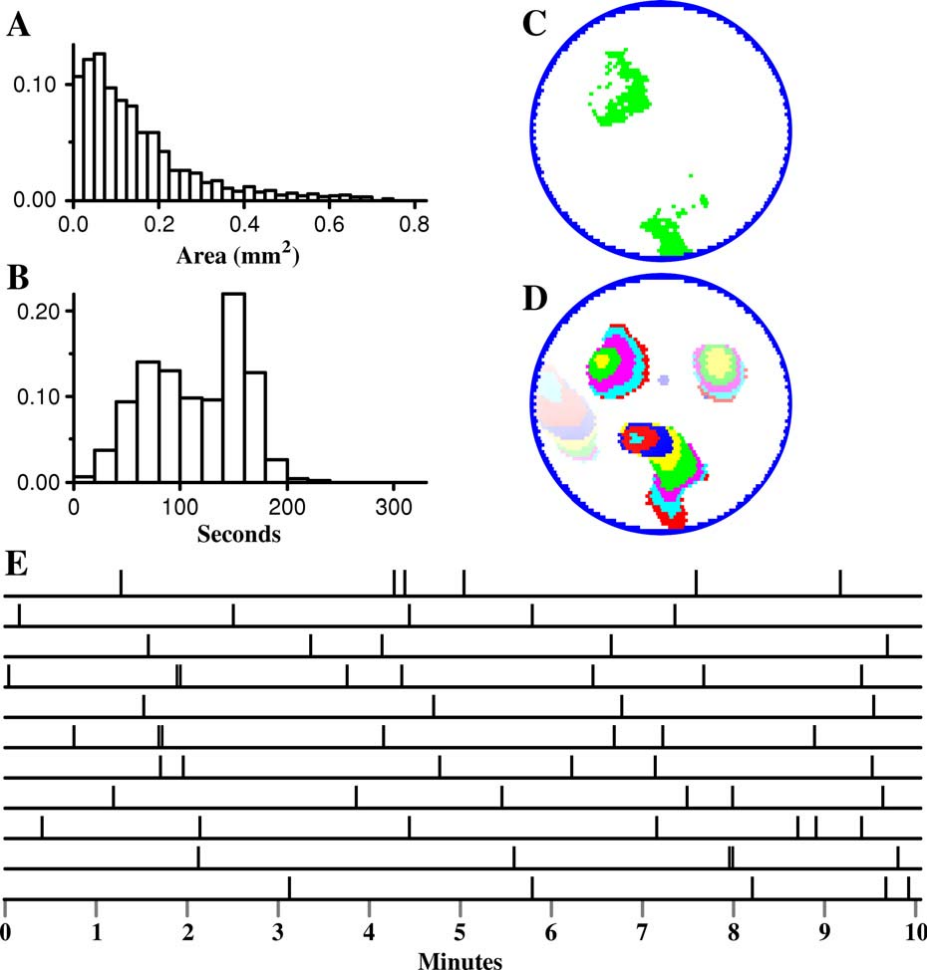
Deterministic Wave Behavior (A,B) Domain size and IWI distributions when the model is run in deterministic mode. (C,D) Wave activity in the amacrine cell layer (instantaneous) and recent wave activity across the retina (15 s; each color represents the advancement of a wave over 500 ms, colors fade to white with time). Results are comparable to Figure 1, suggesting that intrinsic noise is not necessary for the generation of random-looking wave activity. (E) Depolarization times of a single amacrine cell after variation of initial conditions. The last 10 min of a 100 min simulation run are shown. The top line is reference behavior. The following 10 lines show the depolarization times of the same amacrine cell after a different amacrine cell has had its initial threshold value changed by a small amount (∼4%). This change in behavior is representative of all amacrine cells, demonstrating a pronounced collective change in wave behavior as a result of small changes in initial conditions.

Chaotic behavior is generally defined as behavior that is sensitive to small variations in initial conditions and that generates apparent randomness whose origins are entirely deterministic. To investigate this, we initialized the retina as described in Methods and then allowed the state of the model to evolve as a deterministic cellular automaton, where the state at any given time is a strict function of the immediately preceding state, using no added simulated noise. Specifically, *P* (Equation 3) was constant. Analyses showed no significant differences between waves produced in simulations run this way compared to waves generated with ongoing noise. Table 1G shows the parameters used to reproduce P2–P4 ferret waves statistically similar to those measured by [13,14] (Figure 5). The IWI was 115 ± 46 s, average wave velocity was 183 m/s, and wave frequency was 3.0 waves/s/mm^2^. This compares with the physiological IWI of 115 ± 48 s [13], an average velocity of 177 μm/s, and wave frequency of 3.0 waves/s/mm^2^ [14]. The distribution of wave sizes was similar (compare Figures 3A and 5A), with the model producing waves 0.16 ± .16 mm^2^ in size.

To investigate sensitivity to changes in initial conditions, we initialized the retina as above but changed the threshold value of a single amacrine cell by 0.1, which is 4% of the average initial value. We then allowed the model to evolve for 90 min. We repeated this with ten different amacrine cells selected from around the simulated retina and then compared the behavior of a single amacrine (the center cell) across each of these simulations (Figure 5E). The timing and intensity of bursting activity for the observed amacrine cell was different in all tested cases. More notable, however, was the collective change in behavior of all amacrine cells, which produced greatly altered wave patterns when viewing the entire simulated retina. Similar behavior occurs when the model is perturbed while it is running.

To analyze sequential waves and determine if there were any cyclic patterns, we took snapshots of the retina when a wave passed an arbitrarily chosen point and compared sequences of successive waves (Figure 2). No patterns were apparent on the short time scales observed (up to 3 h). To explore the possibility of very long cycle times, we ran several very long simulations (2 or 3 y simulated time) and analyzed model output. To do this, we chose 19 amacrine cells uniformly distributed across the retina, and stored the activity pattern of these cells in a list. For each time step that three or more amacrine cells were active, we created a 19 bit integer, one bit for each cell, with a bit set to “1” if the cell was depolarized and “0” otherwise, and stored this value. After the simulation, we took the final 30 patterns and searched for this sequence in the list. For simulations run this way (*n* = 3), no match was found, indicating that any cycle behavior is longer than biologically relevant time scales. We used a small model retina for this search (0.65 mm^2^, d*T* = 100 ms) on the presumption that a small retina would show shorter cycle times than a large retina. Wave behavior was similar at the beginning and end of this interval both qualitatively and quantitatively (spatiotemporal properties of waves varied by <5%).

The activity-dependent refractory period is critical for the production of non-repeating and apparently random behavior. Analysis showed that the model produced non-repeating wave behavior so long as there was a differential in input received by cells during a wave, and hence a variable increase in their refractory periods. Extending the period where this input was summed (i.e., when the cells refractoriness increased regardless of whether or not it was active) or by reducing the parameter *H*_2_ to very low values produced stable waves which covered the entire retina and eliminated realistic wave behavior. Such changes also eliminate realistic wave behavior when the model is run with stochastic input (i.e., randomly varying *P*).

The observations that the model shows sensitivity to small changes in initial conditions and produces apparently random waves that are non-periodic and with randomly distributed initiation points suggest that it has a deterministically chaotic regime. However, we did not carry out strict mathematical tests for the presence of chaos (calculation of Lyapunov exponents or a complexity analysis of cellular automata [43]), and therefore we do not claim to have demonstrated chaos in a mathematical sense. The model's behavior is consistent with “chaotic aperiodic behavior” as described for cellular automata [43] and is present for all time steps tested (ranging from 5 ms to 200 ms). The biological relevance of this behavior is not that the model is chaotic in a mathematical sense, which would be interesting, but that it is chaotic in a practical sense. It uses a simple mechanism which does not rely on underlying stochasticity to reproduce the non-repeating and random waves that are observed in many species and are mediated by different chemical pathways [11].

### Variable Depolarization Intervals

The model is framed in a simple form in order to focus on general principles behind wave production. One particular simplification regards the duration an amacrine cell depolarizes, which is treated as constant but actually varies, with amacrine cells that depolarize in isolation doing so for relatively brief periods while depolarizations which are coincident with a wave are much longer [30]. To test whether this simplification affected wave production, we modified amacrine cells to have short fixed depolarizations and allowed them to remain depolarized, and hence excitatory, for as long as they had sufficient input from neighboring cells to do so.

**Figure 6:**
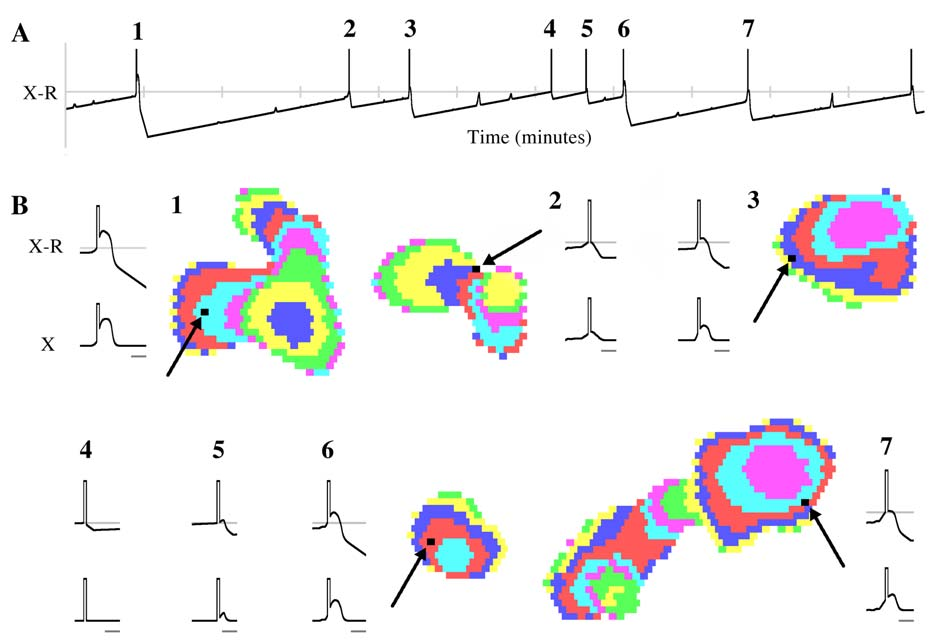
Variable Depolarization Intervals (A) Difference between excitation and threshold of a single amacrine cell shown over an 11 min interval. The cell “depolarizes” and becomes active for a fixed duration (0.45 s) when excitation exceeds threshold (i.e., *X* > *R*; indicated by the vertical spike). The cell remains depolarized and excitatory to its neighbors while *X* > *R*. After each depolarization, the threshold increases as a function of how much excitation the cell received and then slowly decays linearly. (B) Wave activity for each labeled depolarization in (A) is shown along with excitation patterns to the cell. Position of cell in the waves is shown by arrows. The top plot (X-R) is an expansion of that shown in (A) (gray scale bars = 2 s). The lower excitation plot (X) shows the excitation received by the cell during this same period, with the vertical spike indicating the fixed depolarization. Depolarizations 4 and 5 had no corresponding wave activity and were “intrinsic bursts” [30]. While some excitation plots show clear wave activity (1, 3, 6, 7), it is not always possible to distinguish between intrinsic bursts and depolarizing amacrine cells on the outer edges of a wave (compare 2 and 5). Using variable depolarization intervals, approximately 25% of depolarizations were intrinsic bursts.

With this modification, wave behavior still appeared normal, with the model again being able to match the spatiotemporal properties of waves in different species. We targeted reproduction of P2–P4 ferret waves, producing waves with size = 0.16 ± 0.12 mm^2^, velocity = 180 μm/s, IWI = 113 ± 52, and frequency = 3.0 waves/mm^2^/s (model parameters: *H*_1_ = 5.0, *H*_2_ = 0.25, *P* = 36, and *K* = 0.3). While waves appeared normal, amacrine cell behavior was notably different after this modification. Cells firing in isolation produced very brief bursts while amacrine cells that contributed to a passing wave depolarized with a duration and magnitude that varied by the cell's position in the wave (Figure 6).

## Discussion

The main strength of this model is its ability to reproduce the statistical properties of retinal waves seen in several species using only a small set of basic principles consistent with known physiology. Amacrine cells have been observed to spontaneously depolarize [30], they regularly depolarize during wave activity [29,30,44], and they release transmitter when depolarized [45,46], even immediately after passage of a wave [30], thus contributing to wave activity. The model is based on these observations and makes two additional assumptions: that depolarization is increasingly easy to achieve the more time that has elapsed since the previous depolarization, and that cells which receive more input have longer intervals between spontaneous depolarizations. These assumptions are supported by data in a recent study [30] which showed that amacrine cells have slowly decaying afterhyperpolarizing potentials (AHPs), and they have very short refractory periods when pharmacologically isolated, longer refractory periods when they are spontaneously active while connected to neighbors, and even longer refractory periods when they depolarize during a passing wave.

The model presented here differs significantly from previous models of retinal waves [14, 28]. The most recent and related model [14] was based on the assumption of random depolarization of amacrine cells and it required a second layer of RGCs to filter sparse amacrine cell activity to produce wave-like behavior. The present model is based on deterministic activity-dependent refractory periods and produces spatially dense patterns of depolarized amacrine cells. The principle of activity-dependent refractory periods is very general and is not constrained by the properties of any particular neurotransmitter pathway or cell type. It produces waves with a large range of spatiotemporal properties and could underlie the production of waves at many different stages of development, in different species, and even in different brain area. A further difference is that only a single cell layer is required to produce waves, something that was previously not thought to be possible [14]. It should be stressed however, that while the principles we describe are very general, our model only addresses basic wave behavior that occurs in early development and ignores the emerging complexity of the retina as it matures.

There are several ways to implement the basic principles of the model, and we explored some of the possibilities. As described above, amacrine cells were allowed to have both fixed and variable depolarization durations, and the model was run with and without stochastic input. Other strategies that we tested included: producing excitations at random points in the network, as might occur if amacrine cells, or other cells present later in development, were to depolarize spontaneously or to spontaneously release vesicles; using a layer of RGCs to filter amacrine cell activity, similar to [14]; varying the connectivity radius and the connectivity strength; and using continuous (periodic) boundary conditions. None of these variations resulted in significantly different behavior, suggesting that the underlying principles of the model are more important for wave generation than their particular implementation.

In the model, the magnitude of the threshold regulates the refractory period, and this magnitude depends on recent input to the cell, with cells receiving more input during their periods of activity having higher thresholds. The rate of threshold decay was largely constant, resulting in longer refractory periods in cells that contributed to a wave compared to those that depolarized in isolation. Biologically, this refractory period results from a calcium-dependent potassium current [30] and possibly other factors, such as an activity-dependent variation in intracellular chloride, which has been proposed to drive spontaneous activity in the developing spinal cord [47]. The model does not differentiate between mechanisms contributing to the refractory period and only predicts characteristics of the resulting behavior. More physiologically detailed and species-specific models of the retina will be necessary for understanding the finer dynamics of retinal waves, and more experimental data will be required to adequately constrain such models. Given that calcium imaging appears to not detect all wave activity, and the spatial extent of electrode studies is limited, one experiment that would be very helpful would be simultaneous electrode and calcium imaging recordings over retinal areas sufficiently large to discern waves and their boundaries, as this would determine the frequency of very small patches of activity, how much activity is required before Ca^2+^ signal detection is possible, and how focused or extensive actual wave activity is in relation to the calcium imaging responses.

The model produces output that should be useful in computational studies of the developing retino–geniculate pathway [31,33], since the parameters can be adjusted to produce retinal waves with a wide range of size, velocity, and IWI. The output provides a relatively uniform net retinal coverage and it is simple to convert it to RGC spike trains by using amacrine cell activity as input to integrate and fire neurons. However, there are at least two significant ways in which this model does not conform to experimental observation. First, the duration of RGC bursting is weakly explained by the present model, as during a wave amacrine cell activity at a given location in the retina typically lasts 1–3 s. We have made no attempts to reproduce or account for the burst variability seen between species [12], including the seconds-long oscillations of excitation observed in turtle retina [15] or the longer burst times observed in older ferret [11]. The behavior of the model suggests that additional factors are behind the long duration bursts seen physiologically, possibly involving input from additional cell types (e.g., bipolar cells) and the use of metabotropic ion channels and/or additional neurotransmitters (e.g., GABA). Second, simple spiking patterns, as would be produced by integrate and fire neurons, are only seen during early development (but see [48] for integrate and fire neurons which produce various burst patterns). As development proceeds, alpha, beta, and gamma RGCs develop distinct firing patterns [11] and ON and OFF RGCs begin to fire at different rates [49]. Computational studies using the output of this model as input to higher levels of the visual pathway will need to address these factors, as appropriate, according to the particular species and age being modeled.

**Figure 7:**
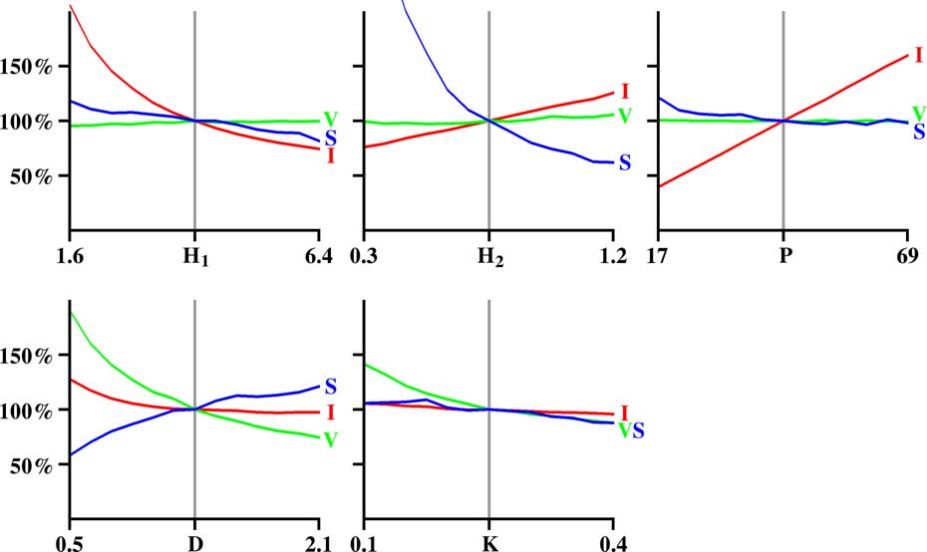
Effects of Parameter Variations on Wave Properties IWI (I), wave size (S), and velocity (V) for different parameters. The vertical gray lines represent the values of parameters from Table 1A (P2–P4 ferret) and serve as a baseline. Parameter values were varied ±60% from baseline and are scaled linearly. Tick marks on the vertical axis represent 50%, 100%, and 150% response versus baseline.

Wave behavior is stable across a wide range of parameter settings (Figure 7). Using Figure 7 as a guide, model parameters can be manipulated to produce waves quite different from those described here, including waves that slowly progress across all cells, or that produce small groups of excitation that propagate very little. Analysis of the effects of changing different parameters show that the duration an amacrine cell is excitatory (parameter *D*) is the most important factor in regulating the velocity of waves, particularly at slower velocities. When simulating mouse waves, it was necessary to increase *D* above 2 s to achieve velocities near the 110 um/s reported physiologically [34]. This suggests that the excitatory mechanism used in these mice involves either a slower excitation, such as would be produced by extracellular diffusion of transmitter, or a reduced rate of transmitter degradation, compared to what occurs in other species. Alternatively, mice may have extended durations of amacrine cell depolarization and/or periods of vesicle release. Wave velocity was similarly affected in simulations where amacrine cells were allowed to depolarize for variable durations. Slowing the onset of the AHP, thus prolonging depolarization, reduced average wave velocity. Increasing the speed of AHP onset increased wave velocity.

A different situation exists in E14–16 chicks, where extraordinarily fast waves are observed (.5–1.5 mm/s) [15,41]. Extremely short duration excitations can produce waves this fast, but the calcium imaging response from such brief depolarizations is greatly attenuated, often below the threshold of detectability. A more natural explanation for the increase in wave velocity is that the excitation time constant (*K*) approaches zero, causing the excitatory influence from one cell to be quickly realized in others. Physiologically, the adenosine/cAMP pathway may be related to the time constant. Adenosine has been shown to enhance transmitter release and to modulate neuronal excitability [50], and both adenosine and cAMP have strong influences on wave velocity [45]. These results suggest that adenosine/cAMP might play a decreasing regulatory role in retinas with higher wave velocities, such as chick.

The model makes several experimentally testable predictions. One is that wave behavior is the result of activity-dependent refractory periods in spontaneously active amacrine cells—normalizing threshold changes, such as by making AHP responses nearly uniform across cells, should eliminate non-repeating waves. Related to this, induction of an activity-dependent refractory period in cells which are recurrently connected and spontaneously active should produce non-repeating wave behavior. A second prediction is that wave velocity should be a function of the duration of excitatory influence of an amacrine cell. Manipulating this interval through genetic or pharmacological means should influence wave velocity. Third, wave behavior, as measured by RGC activity, should spatially extend beyond waves detected through large-scale calcium imaging, meaning that waves as defined by spike activity should be bigger than those seen by calcium imaging.

The commonalities of retinal wave behavior across species and different anatomical and neuropharmacological pathways suggest an underlying mechanism that is robust and capable of being implemented in many ways. Our model displays this flexibility and has been framed to focus on mechanisms likely to be common among species and not to be constrained by specific physiological implementations or specific neural cell types. Hence the principles that we describe may be applicable to the description of activity in other parts of the brain such as auditory system, spinal cord, neocortex, and hippocampus which, like the retina, also exhibit patterns of coordinated spontaneous activity during development [2–12,51].

## Methods

The model focuses primarily on waves mediated by ACh [12,17]. However, the principles behind the model, namely that wave behavior results from spontaneous activity and that refractory periods are a function of recent input, are intentionally general so as to be unconstrained by specific biophysical implementations. Hence, the model is adaptable to the description of waves in several developmental stages and species even though different physiological and pharmacological mechanisms might be responsible for their generation.

The model relies on a single cell type, cholinergic amacrine cells, which are responsible for mediating early (Ach-mediated) retinal waves [27]. Amacrine cells in the model are spontaneously active and form excitatory connections with other nearby amacrine cells. Here, “active” means depolarized and/or actively exciting its neighbors. These cells have a varying threshold for activation which is high immediately after depolarization and gradually decays, similar to, but slower than, the change in spike threshold following a spike in a normal neuron. The magnitude of the threshold change is a function of recent input to the cell: a cell receiving more input has a higher threshold immediately after depolarization. When the cell's threshold decays to zero, or when its level of excitation exceeds its current threshold, it depolarizes and becomes active, exciting neighboring amacrine cells. When a sufficient density of amacrine cells becomes active, local excitation brings other nearby amacrine cells to threshold, producing a wave of excitation. Amacrine cell activity propagates as long as there is an adequate density of nearby amacrine cells close enough to threshold to depolarize from the excitation of the advancing wave front.

**Figure 8:**
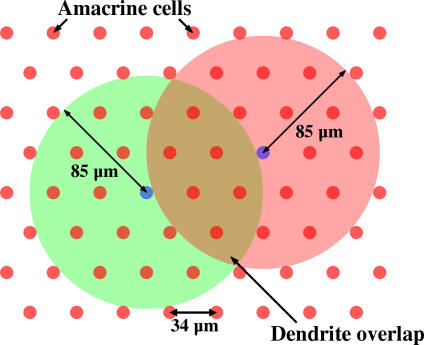
Network Topography Amacrine cells are arranged in a triangular lattice with a distance between cells of 34 μm. The dendrite of each amacrine cell extends 85 μm from the soma, and the excitatory coupling between two cells is proportional to the area of overlap of their dendrite arbors.

The model retina is circular and its amacrine cells are arranged in a triangular lattice (Figure 8). Based on the measured cell density of 1,000 starburst amacrine cells per mm^2^ [13], the distance between adjacent amacrine cells was estimated to be 34 μm. A dendrite radius of 85 μm was used, midway between observations reported from different studies in newborn rabbit [30,32]. The excitatory strength between amacrine cells was proportional to the area of overlap of dendritic arbors. On initialization, the location of amacrine cells was precomputed, and all nearby cells having an overlapping dendritic arbor were stored in a list (set *Z_i_*)

**Figure 9:**
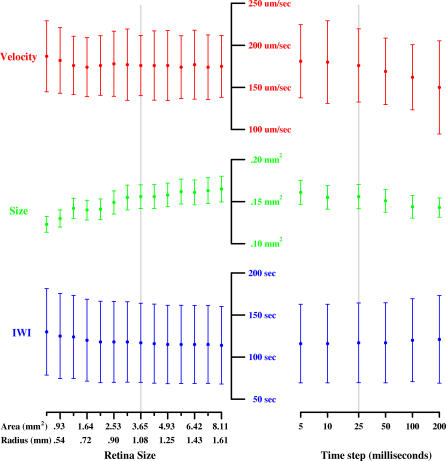
Variations of Simulation Time Step and Retina Size on Wave Properties Variations in wave velocity, size, and frequency for retinal sizes between 0.65 and 8.1 mm^2^ (left) and simulation time steps between 5 ms and 200 ms (right; horizontal axis on log scale). Except as otherwise noted, simulations were based on a retinal size of 3.65 mm^2^ and used a time step of 25 ms (indicated by vertical gray lines). Mean (closed circles) and standard deviation (vertical bars) are shown. The model is stable across a wide range of simulation time steps and retinal sizes.

To determine a suitable size for our model retina, we explored the spatio–temporal properties (velocity, size, and IWI) of simulated P2–P4 ferret waves for model retinas between 0.65 mm^2^ and 8.11 mm^2^ in size. Wave behavior varied across this range but was stable, with wave size showing the most variation (Figure 9). The smallest retina tested (0.65 mm^2^) produced waves which were 27% smaller than those seen on the largest retina (8.11 mm^2^). For computational speed, we selected a retinal size of 3.65 mm^2^, which produced waves with measured properties within 5% of those produced on the largest retina. Similarly, we explored different time steps for the model (Figure 9). For the simulations, we used a time step (Δ*T*) of 25 ms except as otherwise noted, which produced waves with measured properties (velocity, size, and IWI) within 5% of those produced with the smallest time step tested (5 ms).

The input, *N_i_*, to each amacrine cell was the weighted sum of all nearby and active amacrine cells:

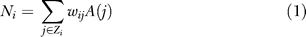
where *w_ij_* is the excitatory strength between amacrine cells *i* and *j*, and *A*(*j*) is the output of amacrine cell *j*. *A*(*j*) = 1 for an amacrine cell that was active in the previous time step (i.e., depolarized) and 0 otherwise. The value *w_ij_* equals the relative area of dendrite overlap between the two cells (i.e., the area of overlap divided by the total area of a dendrite arbor).

The excitation level of each cell, *X_i_*, approached the current level of input to that cell at a rate

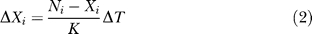
where *K* is the time constant. Whenever *X_i_* exceeded the firing threshold, the cell was said to become active. Each cell maintained an independent threshold, *R_i_*, which was time-varying. When *X_i_* > *R_i_*, the amacrine cell became active for a duration *D*. After this interval had passed, *X_i_* was reset to zero. Threshold, as used here, is not to be taken literally. It is meant to reflect the increase in excitatory input required for a cell to become active (i.e., depolarize) and includes factors such as the AHP [30] and activity-dependent changes to chloride concentrations [47].

Each cell's threshold slowly decreased and the cell spontaneously depolarized when *R_i_* reached zero. The parameter *P* represented the length of time between spontaneous depolarizations for a cell receiving no external input. When a cell was depolarized (i.e., *A*(*i*) = 1), its threshold also increased by a constant amount plus a function of input received. The threshold changed according to:

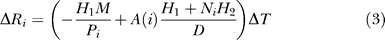
where *H*_1_ is a fixed rate of threshold increase and *H*_2_ is the incremental change in threshold, whose contribution varied based on how much input a cell had. *D* is the depolarization duration of the cell and *M* is a normalizing factor equal to the maximum excitatory input to a cell divided by the maximum excitatory input of a cell in the center of the retina. *M* = 1.0 for the entire retina except the border regions, where amacrine cells have dendrites that extend beyond the retinal boundary, thus having fewer innervating cells. This reduced input results in border cells having smaller increases in *R_i_* after activation and so requires slower rates of decrease in order to help normalize the frequency of spontaneous activations between central and border regions. The incremental change in threshold (*N_i_H*_2_) produces a larger change of threshold for amacrine cells that are in the central region of a wave compared to those near the edge or that depolarize in isolation. It is this differential in threshold behavior that is most critical for generation of finite non-repeating waves.

The model was initialized by assigning each *R_i_* a uniform random value selected from the interval (0.5, 5.0). Because this is unlikely to represent anything achieved during normal activity, the model retina was allowed to run for a time in order to achieve a stable operating state. This was defined as a state where, for a given set of parameters, mean IWI and domain size changed 10% or less compared to a run of 5 h simulated time. In all cases measured, 30 simulated min was sufficient to reach this state. A 1 h initialization period was then used as a “warmup” period for all simulations to further minimize any possible influence of starting conditions. Unless otherwise indicated, all simulations were run for 180 min following the warmup period. Various methods of model initialization were explored (all involving assignment of *R_i_*). All methods tested reached a stable state within several hours of simulated time with the exception of the trivial symmetric case where all *R_i_* were equal. Initializing all *R_i_* to be equal except for one cell, used to break symmetry, was sufficient to produce stable waves after several hours, even when the model was run deterministically (i.e., no “noise”, see below). The initialization method described above was selected as it provides minimal initial bias and approaches a stable state reasonably quickly. Except where explicitly noted, references to time in this study refer to simulated time, not model execution time.

To introduce “noise” into the model, *P* was varied among cells and with each depolarization, producing variability in the interval between spontaneous depolarizations. To do this, *P* was multiplied by a normal random variable with mean of 1.0 and standard deviation of 0.2, to give a cell-specific value, *P_i_*, used for the calculation of *ΔR_i_*. This value for *P_i_* was used by the cell until its next activation, when a new value was chosen. The model was also run as a strict cellular automaton where no randomness was introduced to the model beyond the initial starting conditions.

The free parameters of the model are *P*, which regulates the interval between spontaneous amacrine cell depolarizations; *H*_1_ and *H*_2_, which control the increase in threshold after an amacrine cell depolarizes; the depolarization interval *D*, during which an amacrine cell actively excites its neighbors, and the time constant *K*, which regulates how fast cells react to excitation from neighboring cells. Table 1 lists parameter sets used to produce waves similar to those seen in different species. Parameters *P*, *D*, and *K* are in units of seconds.

Most simulations were performed on a 2.4 GHz Core 2 Duo desktop running linux (Ubuntu 7.04). The model was implemented in C++ and most simulation times varied between 1 and 15 real-time min when running the model for 180 simulated min, the variable time depending on the retina size, the time step used, and the level of amacrine cell activity. For analysis, amacrine activity was saved to file and Java-based tools were used to analyze wave properties. Parameter exploration on some alternate implementations of the model was performed on a Beowulf cluster supercomputer. Source code (C++ and Java), including applets for viewing waves, can be downloaded from http://swindale.ecc.ubc.ca/retinalwaves/.

### Data analysis.

**Figure 10:**
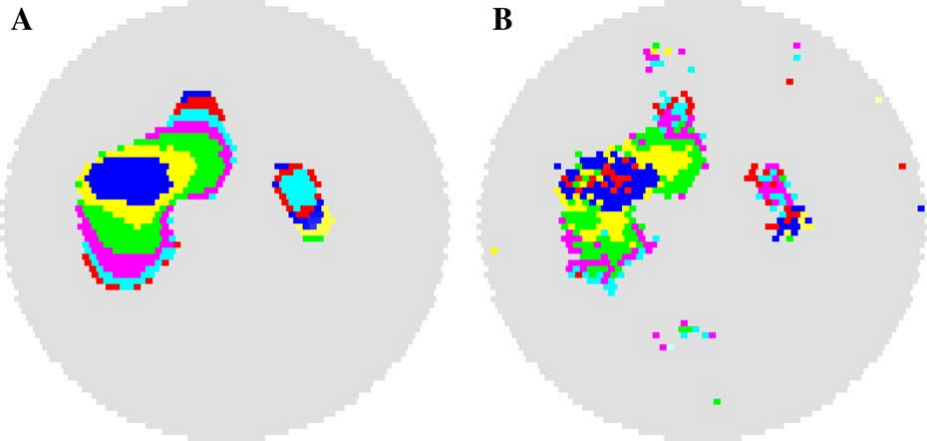
Effects of Smoothing Behavior on Waves Simulated calcium imaging smoothes wave boundaries but does not change the overall shape of waves. (A) Recent (15 s) wave activity as measured through simulated calcium imaging. Different colors show the advancement of a wave over 500 ms intervals. (B) Amacrine cell activity measured over the same interval and using an identical color scheme. The timing of amacrine cell depolarizations differs slightly from the observed wave seen through calcium imaging but wave boundaries are very similar. The small islands of activity in (B) are amacrine cells undergoing spontaneous depolarizations that are not part of wave activity (comparable to “intrinsic bursts” as reported in [30]).

In order to better compare amacrine cell behavior to the experimental results of calcium imaging studies, a rough approximation of a calcium response was produced and the spatial patterns of active (i.e., depolarized) amacrine cells were measured. The retina was partitioned into pixels, one for each amacrine cell, with each pixel assigned a luminance value based on the activation level of all cells with dendrites passing through that point in the retina. Each pixel operated as a leaky integrator and had an intensity calculated according to

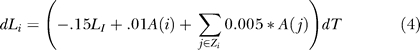
where *L* is the pixel intensity at pixel *i* (bounded on [0,1]), *j* is a set of all amacrine cells with dendrite overlapping *i*, and *dT* = 100 ms. All dendrites passing through a point on the retina contribute to the calcium response, with the soma generating a stronger response than the dendrite. Because of the short dendritic spread of RGCs [52], the addition to the calcium signal due RGCs should have minimal effect on the spatial dynamics of the signal. This transformation is a coarse approximation and was not required for the model to produce wave behavior. It was primarily used to smooth wave progression and wave boundaries, assisting in automated wave tracking, and also to make model output resemble the experimental results more closely (Figure 10). The wave propagation images in Figures 1, 2, 5, and 6 are based on simulated calcium imaging.

A wave was detected when a) the luminance of a pixel exceeded a threshold (*L* ≥ 0.30); and b) the pixel was not adjacent to any pixels that were assigned to a pre-existing wave. The pixel was considered to be part of a wave until its value fell below a lower threshold (*L* < 0.25). This threshold range was used to minimize pixels near threshold from repeatedly joining a wave when oscillating near threshold. The initialization point of this wave was the centroid of all connected pixels which exceeded the lower detection threshold (0.25) on the first frame the threshold was crossed. Wave velocity was calculated by measuring the distance between the centroid and the farthest point reached by the wave and dividing by the time required to reach that point. The velocity of each wave was stored, and the average of these values calculated. When two waves collided, both were omitted from the calculations as there was no longer an unambiguous starting or most distal point. Analysis did not demonstrate any significant difference between these joined waves and waves which remained independent, so their exclusion should not significantly bias the measurements. Wave size was calculated using the number of connected pixels that crossed threshold during the lifespan of the wave (each pixel was counted only once).

To reduce border effects in IWI and retinal coverage calculations, pixels associated with amacrine cells within one dendritic radius of the retinal boundary were omitted from the analysis. The IWI distributions were calculated by measuring the inter-wave interval of all analyzed pixels and storing these values in a histogram.

### Variable duration depolarization.

The model is framed in the simplest form we found that produced robust wave behavior. One simplification involved the duration of amacrine cell depolarizations, which was constant in the model, although studies show that it varies, depending on whether the cell depolarizes in isolation or contributes to a wave [30]. To explore if our simplified amacrine cell behavior affected wave production, we modified amacrine cells by allowing them to depolarize for brief fixed intervals and remain depolarized as long as sufficient excitation was present. This was done by (a) slowing the onset of threshold increase, where the factors governing the refractory period, such as the AHP, take seconds to be fully realized, thus allowing prolonged depolarizations to occur, and (b) not resetting the excitation level (*X_i_*), allowing it to always reflect current input to the cell. As long as excitation was greater than the threshold, the amacrine cell was depolarized and excitatory to its neighbors. A 3 s refractory period was imposed after each fixed depolarization interval to prevent multiple triggerings during a single wave. In these simulations, *D* was set to 0.45 s and the maximum change of threshold (*ΔR_i_*) per second was limited to 4.0. Larger threshold changes took more than 1 s to be fully realized. Other values for *D* and threshold onset rates were explored and produced similar results.

## Supporting Information

Video S1 Four Minutes of Wave Activity on a Retina with Periodic Boundary Conditions Depolarized amacrine cells are white. The color palette for non-depolarized cells shows each cell's difference between excitation and inhibition (X-R; see Figure 1). Cells that are strongly refractory are blue/black, while cells near the point of depolarization are yellow/orange. The simulation in this video uses parameters for ferret waves (Table 1A).
(8.3 MB AVI)

## Abbreviations

AHP: afterhyperpolarizing potential
IWI: interwave interval
RGC: retinal ganglion cell
